# Triple negative breast carcinoma is a prognostic factor in Taiwanese women

**DOI:** 10.1186/1471-2407-9-192

**Published:** 2009-06-18

**Authors:** Che Lin, Su-Yu Chien, Li-Sheng Chen, Shou-Jen Kuo, Tsai-Wang Chang, Dar-Ren Chen

**Affiliations:** 1 Comprehensive Breast Cancer Center, Changhua Christian Hospital, Changhua, Taiwan; 2 Department of Pharmacology, Changhua Christian Hospital, Changhua, Taiwan; 3 Department of General Surgery, National Cheng Kung University Hospital, Tainan, Taiwan

## Abstract

**Background:**

Currently, there is a debate as to whether triple negative breast carcinoma (TNBC) has a worse prognosis than non-TNBC. Our aim was to determine whether TNBC is a prognostic factor for survival.

**Methods:**

We identified 1,048 Taiwanese breast carcinoma patients, of whom 167 (15.9%) had TNBC. Data used for analysis were derived from our cancer registry database for women with breast cancer who were diagnosed between 2002 January and 2006 December.

**Results:**

In the Kaplan-Meier analysis, tumor subgroup (TNBC vs. non-TNBC) was a prognosis factor related to 5-year overall survival. In the univariate analysis, tumor subgroup (TNBC vs. non-TNBC) was a significant factor related to 5-year overall survival, in addition to age, tumor size, lymph node, metastasis, grade, stage, estrogen receptor status, progesterone receptor status, and HER2 overexpression status. In the multivariate analysis, tumor subgroup was not a significant factor related to 5-year disease-free survival (DFS). In node-positive patients, tumor subgroup was a significant factor related to 5-year overall survival, in addition to age, tumor size, metastasis, and grade. In node-negative patients, tumor subgroup was not a significant factor related to 5-year disease-free survival and 5-year overall survival.

**Conclusion:**

Our results indicated that TNBC patients in Taiwan have worse 5-year overall survival than non-TNBC patients. Notably, in node-positive patients, TNBC played a prognostic role in 5-year overall survival.

## Background

Many oncologists think breast cancer is a clinically heterogeneous disease with different responses to treatment and outcomes [1,2]. Sixty to 80% of tumors are positive for the estrogen receptor (ER) and/or progesterone (PgR), and 20% to 40% have her2/neu (HER2) gene over-expression [1]. Interestingly, some recent data suggested that triple negative breast carcinomas (TNBC), ER-negative, PgR-negative and HER2-negative exhibited different clinical outcomes [3,4]. However, there is uncertainty about the appropriate survival role for TNBC. Information on the TNBC subtype is still limited and confusing in adjuvant chemotherapies [4-15]. Liedtke revealed that patients with TNBC have increased pathologic complete response rates (pCR) compared with non-TNBC patients, and those with pCR have excellent survival [3]. Liedtke also demonstrated that patients with residual disease after neoadjuvant chemotherapy have significantly worse survival if they have TNBC compared with non-TNBC, particularly in the first 3 years. Because these studies were done in the other countries, their findings might not apply to Taiwan. In this study, we sought to determine the risk associated with TNBC in Taiwan.

Therefore, the aim of this study was to determine the prognostic significance of TNBC with respect to disease-free survival (DFS) and overall survival in a group of homogeneously-treated Taiwanese breast carcinoma patients.

## Methods

Patients were identified from the databases of the cancer registry at Changhua Christian Hospital, which is located in central Taiwan. Data collection for cancer in this medical center began in 1986 and continued until 2009. The well-trained case managers used the registry software and collected uniform information about all patients with breast cancer who had been examined at least once as outpatients or inpatients in the daily clinical service. This study was approved by the institutional review board of Changhua Christian Hospital (IRB number: 080325). The baseline data included demographic characteristics (e.g., age), tumor characteristics (e.g., tumor size, positivity of lymph node, metastasis, grade, pathologic stage, ER/PgR/HER2 information and histology). Patients with ductal carcinoma in situ only were excluded. The data underwent a variety of editing checks and procedures, so as to omit duplicate records. The quality of the cancer registry database was reviewed and approved by the committee, which consisted of radiologists, oncologists, pathologists, surgeons and epidemiologists with special expertise in breast cancer.

Tumor size was determined on the basis of pathological reports from the Changhua Christian Hospital. The Bloom-Richardson grading system was used for tumor grading. This grading scheme is based on three morphologic features: degree of tumor tubule formation, tumor mitotic activity, and nuclear pleomorphism of tumor cells. Seven possible scores are condensed into three Bloom-Richardson grades: I, II, or III. Staging in this study was presented by the American Joint Committee on Cancer stage group.

Immunohistochemistry (IHC) analysis was performed on formalin-fixed, paraffin-embedded breast cancer tissue. The ER and PgR analysis was based on a IHC assay, in which a report of 10% or greater of cells that had nuclear staining for ER was considered a positive result as well as PgR. IHC was performed with anti-ER (NeoMarkers, clone: SP1, dilution: 1:200, Fermont, California) and anti-PgR antibody (NeoMarkers, clone:SP2, dilution: 1:250, Fermont, California) by an autostaining system (Ventana Medical Systems, Tucson, Arizona).

Breast cancer tumors were classified as HER2-positive if they demonstrated HER2 gene amplification using the fluorescence in-situ hybridization method, or were scored as 3+ by an IHC method. HER2 IHC only used cell membrane localization to interpret (Dako, Carpinteria, California). The intensity of the membrane staining was defined by a semiquantitative score (0 to 3+). Tumor staining was compared to staining of normal breast epithelium from the same patient as a negative control. For clinical purposes, 3+ staining, defined as uniform and intense membrane staining in more than 30% of invasive breast cancer cells, was considered overexpression. No staining or weak incomplete membrane staining was considered a negative result.

Data used for analysis were derived from the cancer registry database of women with breast cancer who were diagnosed between 2002 January and 2006 December. Data for analysis started from 2002, because there was a lack of information on HER2 in patients before 2002. DFS was defined as freedom from breast cancer recurrence or breast death. Overall survival was defined as freedom from breast cancer death or other causes of death.

Postoperative adjuvant therapy has been performed based on the recommendation of NCCN or St. Gallen guidelines with anthracycline-based regimens (in this study mostly FEC therapy: 5-FU 500 mg/m^2^, epirubicin 75–90 mg/m^2^, cyclophosphamide 500 mg/m^2^) as chemotherapy. Taxanes (paclitaxel, docetaxel) were added to follow FEC therapy in few high risk patients.

The patient group included 1,048 females with an average age of 51.8 years (standard deviation [SD] = 11.9 years). Data are expressed as mean ± SD for continuous variables. Independent t tests were used for the comparison of continuous variables. Categorical variables were normally tested by the χ^2 ^test when appropriate. All *p *values are two-tailed; a *p *value of less than 0.05 was considered to indicate statistical significance.

We used Cox proportional hazard analysis to assess the risk of recurrence or mortality relative to the prognostic factors in breast cancer cases. Cumulative survival rates of breast cancer cases were analyzed by the Kaplan-Meier method. The differences of cumulative survival were assessed using the log-rank method. All statistical analyses were performed with SAS 9.1 software.

## Results

Eighty-two (7.8%) patients died for cancer-related reasons during their follow-up, up to 31, December 2007. Five (0.48%) patients died for non-cancer-related reasons. The average follow-up time was approximately 40 months. One hundred and sixty-seven patients (15.9%) had TNBC and the remaining 881 patients (84.1%) were defined as non-TNBC. There was no distributional difference of adverse prognostic factors between the two groups, except lymph node, and grade (Table 1). Seventy-one cases with recurrence of breast cancer were considered as the events.

**Table 1:** Descriptive statistics of women with breast cancer according to tumor subgroup

Features		Total (n)	Tumor subgroup		*p*-value
			TNBC (n = 167)	non-TNBC (n = 881)	
Age, years (SD)			51.97 (13.04)	51.73 (11.79)	0.81
Tumor size, cm (%)					
	<2.0	446	46.1	46.3	0.9651
	≥2.0	517	53.9	53.7	
	Other or Unknown	85			
Lymph node (%)					
	Negative	553	70.1	54.8	0.0004*
	Positive	413	29.9	45.2	
	Other or Unknown	82			
Number of axillary lymph nodes involved (n)					
	1–3	210	45.7	51.8	0.6848
	4–9	122	34.8	29.0	
	10+	79	19.6	19.2	
Average number of axillary lymph nodes involved per node-positive patient			6.17 (5.91)	6.19 (7.92)	0.99
Metastasis (%)					
	No	938	96.8	97.8	0.4781
	Yes	23	3.2	2.2	
	Unknown	87			
Metastasis site (n)					
	Bone	9	1	8	
	Lung	1	1	0	
	Liver	2	0	2	
	Other	2	0	2	
	Multiple	5	1	4	
	Unknown	4	2	2	
Grade (%)					
	I ~II	693	43.4	73.8	<0.0001
	III	311	56.6	26.2	
	Unknown	44			
Stage					
	< II	315	35.1	32.7	0.5622
	≥ II	638	64.9	67.3	
	Unknown	95			
Estrogen receptor (ER)					
	Negative	369	100	23	
	Positive	675	0	77	
Progesterone receptor (PR)					
	Negative	383	100	25	
	Positive	661	0	75	
Her2/neu gene (HER2) overexpression					
	Negative	789	100	71	
	Positive	259	0	29	
Histological types					
	Infiltrating duct carcinoma, NOS	939	91.6	89.2	
	Lobular Carcinoma, NOS	24	0	2.7	
	Mucinous adenocarcinoma	24	0	2.7	
	Infiltrating duct mixed with other types of carcinoma	18	1.8	1.7	
	Others	43	6.6	3.6	

The univariate analysis for prognostic factors associated with 5-year DFS revealed that the tumor group, whether TNBC or non-TNBC, as well as age and menopausal status, was not statistically significant (Table 2). The univariate analysis for prognostic factors associated with 5-year overall survival revealed that the tumor group as TNBC or non-TNBC was statistically significant, in addition to age, tumor size, lymph node, metastasis, grade, stage, ER status, and PgR status (Table 2).

**Table 2:** Prognostic factors for 5-year disease-free survival (DFS) and overall survival in univariate Cox regression analysis

Features	DFS			Overall Survival		
		
	HR	95% CI	*p*-Value	HR	95% CI	*p*-Value
Age	1.00	0.99–1.01	0.9859	1.03	1.01–1.04	0.0018
Menopausal status						
(Postmenopausal vs. Premenopausal)	1.04	0.76–1.42	0.8132	1.51	0.98–2.33	0.0623
Tumor size, cm	1.22	1.16–1.29	<0.0001	1.20	1.11–1.31	<0.0001
Lymph node						
(Positive vs. Negative)	3.08	2.14–4.42	<0.0001	2.83	1.71–4.68	<0.0001
Metastasis						
(Yes vs. No)	4.25	2.22–8.11	<0.0001	9.40	4.78–18.49	<0.0001
Grade						
(III vs. I ~II)	1.71	1.23–2.38	0.0014	2.03	1.31–3.16	0.0017
Stage						
(≥ II vs. < II)	3.03	1.87–4.94	<0.0001	2.53	1.33–4.84	0.0048
ER status						
(Negative vs. Positive)	2.26	1.64–3.12	<0.0001	3.08	1.98–4.79	<0.0001
PgR status						
(Negative vs. Positive)	1.95	1.40–2.70	<0.0001	2.35	1.50–3.68	0.0002
HER2 overexpression status						
(Negative vs. Positive)	1.97	1.41–2.75	<0.0001	1.56	0.99–2.46	0.058
Tumor subgroups						
(TNBC vs. non-TNBC)	1.43	0.99–2.06	0.0570	1.99	1.26–3.13	0.0031

The multivariate analysis for prognostic factors associated with 5-year DFS revealed that the tumor group as TNBC or non-TNBC was not statistically significant. The multivariate analysis for prognostic factors associated with 5-year overall survival revealed that the tumor group as TNBC or non-TNBC was not statistically significant (Table 3).

**Table 3:** Prognostic factors for 5-year DFS and overall survival in multivariate Cox regression analysis

Features	DFS			Overall Survival		
		
	HR	95%CI	*p*-Value	HR	95%CI	*p*-Value
Age	1.00	0.98–1.01	0.9058	1.04	1.01–1.06	0.0013
Tumor size, cm	1.19	1.10–1.28	<0.0001	1.17	1.04–1.32	0.0110
Lymph node						
(Positive vs. Negative)	2.65	1.79–3.92	<0.0001	2.36	1.35–4.12	0.0026
Metastasis						
(Yes vs. No)	2.30	1.15–4.64	0.0193	6.63	3.13–14.09	<0.0001
Grade						
(III vs. I ~II)	1.54	1.05–2.26	0.0269	2.11	1.23–3.62	0.0065
Tumor subgroups						
(TNBC vs. non-TNBC)	1.28	0.82–1.99	0.2835	1.66	0.93–2.96	0.0881

**Node-positive**						

Age	0.99	0.97–1.01	0.2960	1.04	1.01–1.07	0.0161
Tumor size, cm	1.23	1.10–1.37	0.0002	1.29	1.11–1.51	0.0011
Metastasis						
(Yes vs. No)	2.37	1.15–4.88	0.0198	7.52	3.37–16.79	<0.0001
Grade						
(III vs. I ~II)	1.49	0.93–2.36	0.0948	2.46	1.23–4.90	0.0109
Tumor subgroups						
(TNBC vs. non-TNBC)	1.13	0.63–2.03	0.6847	2.20	1.08–4.49	0.0296

**Node-negative**						

Age	1.02	0.99–1.04	0.1801	1.04	1.01–1.07	0.0186
Tumor size, cm	1.16	1.02–1.31	0.0236	1.00	0.74–1.36	0.9813
Metastasis*						
(Yes vs. No)	-	-	-	-	-	-
Grade						
(III vs. I ~ II)	1.49	0.75–2.94	0.2526	1.69	0.69–4.12	0.2525
Tumor subgroups						
(TNBC vs. non-TNBC)	1.52	0.75–3.06	0.2461	1.28	0.47–3.19	0.6815

* Patients without metastasis were censor observations by the end of study period

**Figure 1:**
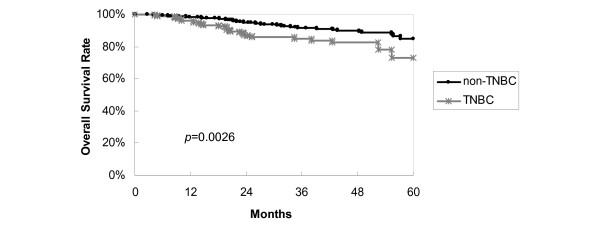
**Overall survival curves by tumor subgroup**. Triple negative breast cancer (TNBC) tended to display a worse 5-year overall survival (*p *= 0.0026) than non-TNBC, by log-rank analysis.

**Figure 2:**
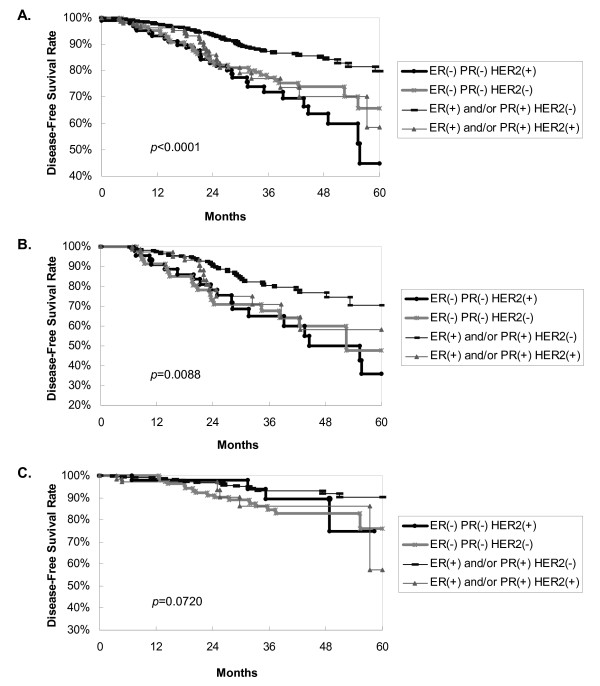
**Disease-free survival (DFS) curves according to patient group**. **A**, among 1,048 patients, ER-negative and PgR-negative and HER2-positive breast carcinoma tended to display the worst 5-year DFS, by log-rank analysis; ER-positive and/or PgR-positive and HER2-negative patients had the best 5-year overall survival; *p *< 0.0001. **B**, in the node-positive patients, TNBC tended to display a worse 5-year DFS by log-rank analysis; ER-positive and/or PgR-positive and HER2-negative patients had the best 5-year DFS; *p *= 0.0088. **C**, in the node-negative patients, four subgroups showed no 5-year DFS difference by log-rank analysis; *p *= 0.0720. We followed up the node-negative HER2-positive patients up to 48 months. **ER**, estrogen receptor; **PgR**, progesterone receptor; **HER2**, her2/neu gene over-expression.

**Figure 3:**
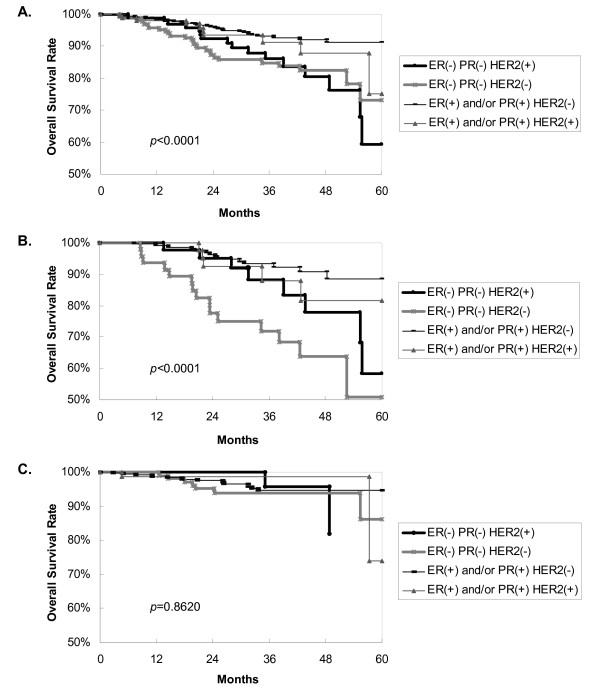
**Overall survival curves according to patient group**. **A**, among 1,048 patients, HER2-positive breast carcinoma tended to display the worst 5-year overall survival by log-rank analysis; our ER-positive and/or PgR-positive and HER2-negative patients had the best 5-year DFS; *p *< 0.0001. **B**, in the node-positive patients, TNBC tended to demonstrate the worst 5-year overall survival by log-rank analysis; ER-positive and/or PgR-positive and HER2-negative patients had the best 5-year overall survival; *p *< 0.0001. **C**, in the node-negative patients, four subgroups showed no 5-year overall survival difference, by log-rank analysis; *p *= 0.8620. We followed up the node-negative HER2-positive patients up to 48 months.

Survival curves are shown in the figures. Figure 1 reveals that TNBC tends to display a worse 5-year overall survival (*p *= 0.0026) than non-TNBC, using by log-rank analysis. ER-positive and/or PgR-positive and HER2-negative patients had the best clinical outcome, with a 5-year DFS of 80%; ER-negative, PgR-negative and HER2-positive patients (HER2-positive subtype) showed the worst outcome with a 5-year DFS of 45% (Figure 2A). TNBC has relatively poor prognosis. Figure 3A shows ER-positive and/or PgR-positive and HER2-negative patients had the best clinical outcome, with a 5-year overall survival of 91%, and the HER2-positive subtype showed the worst outcome, with a 5-year overall survival of 59%.

To investigate the lymph node factor, we analyzed node-positive and node-negative patients individually. For node-positive patients, Figure 2B demonstrates that TNBC breast carcinoma tends to show a worse 5-year DFS, by log-rank analysis, and that ER-positive and/or PgR-positive and HER2-negative patients have the best 5-year DFS (*p *= 0.0088). For node-negative patients, Figure 2C reveals that the four subgroups do not show a 5-year DFS difference, using log-rank analysis (*p *= 0.0720).

For node-positive patients, Figure 3B points out that TNBC breast carcinoma patients tend to have the worst 5-year overall survival, by log-rank analysis, and that ER-positive and/or PgR-positive and HER2-negative patients have the best 5-year overall survival (*p *< 0.0001). For node-negative patients, Figure 3C indicates that the four subgroups do not show a 5-year overall survival difference, by log-rank analysis (*p *= 0.8620).

## Discussion

We present the results of the largest Taiwanese study to date that thoroughly investigates the clinical phenotype of TNBC with regard to DFS and overall survival.

Traditionally, breast carcinomas have been classified as hormone receptor-positive or negative. Recently, newer approaches to breast carcinoma classification using gene-expression profiles and IHC biomarkers have identified at least four subtypes [10]. These subtypes are, luminal A (ER-positive and/or PgR-positive and HER2-negative), luminal B (ER-positive and/or PgR-positive and HER2-positive), basal-like (ER-negative, PgR-negative and HER2-negative; mostly TNBC) and HER2-positive (ER-negative, PgR-negative and HER2-positive). ER- and PgR-negative tumors are generally thought to have a poor prognosis because of a deficiency of hormone therapy strategies. HER2-negative tumors lack the benefit of HER2-targeted therapy and are thought to imply a worse prognosis, as well [10]; however, few TNBC data have been reported among non-Western populations. Our data showed that TNBC in Taiwan may have a different meaning than in Western countries.

Table 1 revealed TNBC subgroup had higher rates of node-negative cases, in agreement with comparable studies [4,12,16,17]. Some series had different results from ours [3,6,7,10,13,14,18,19].

Our univariate Cox regression analysis demonstrated that tumor size, lymph node status, metastasis, grade, stage, ER status, PgR status, and HER2 status except tumor TNBC subgroup were the prognostic factors for 5-year DFS, in contrast to comparable series [4,6,17]. There were discrepant findings between the outcomes of DFS and overall survival in our data. In CALGB 9344 trial, taxanes demonstrated a statistically significant improvement in DFS but not overall survival. It may interpret the discrepancy. In this study the chemotherapy regimen of most patients was FEC therapy. Due to Taiwan national health insurance policy, using FEC followed by Taxanes was only allowed in node-positive and ER negative breast cancer women. In Taiwan node-positive TNBC patients were allowed to receive Taxanes which improved DFS but not overall survival. Furthermore, Yin et al showed 85.1% of their patients were administered adjuvant chemotherapy of different regimens for 4–6 cycles [4]. Mersin said their patients had adjuvant chemotherapy according to the current guidelines at that time [6]. Fulford et al. reported DFS and overall survival results that were different from ours. Their study demonstrated that basal tumors, mostly TNBC, exhibited a significantly better DFS and overall survival than non-basal tumors; however, the study was based on 470 patients with grade III invasive ductal carcinomas diagnosed between 1975 and 1991. Twenty six percent of the patients received adjuvant chemotherapy [17].

Our multivariate Cox regression analysis demonstrated that tumor subgroup (TNBC vs. non-TNBC) was not the prognostic factor for 5-year overall survival These findings were in contrast with those of previous studies [3,5,12-14,20]. Liedtke reported that decreased 3-year overall survival was observed for patients with TNBC, compared with non-TNBC (3-year overall survival rates: 74% vs. 89%; HR = 2.53; 95% CI, 1.77 to 3.57; *p *< 0.001) [3]. In addition, our results are consistent with those from other studies with respect to prognostic factors for overall survival [16]. Kim reported that basal-like carcinoma, mostly TNBC, did not show survival difference between other subgroups, except the HER2-positive subgroup.

To further study the lymph node factor, we stratified patients into node-positive and node-negative groups. In node-positive patients, TNBC breast carcinoma tended to demonstrate the worst 5-year overall survival by log-rank analysis (Figure 3B), and ER-positive and/or PgR-positive and HER2-negative patients had the best 5-year overall survival (*p *< 0.0001). Figure 3C shows four subgroups of node-negative patients that did not reveal a 5-year overall survival difference by log-rank analysis (*p *= 0.8620). Our results are in contrast to those of a previous study by Rakha, which reported that both univariate and multivariate analyses showed basal phenotype, mostly TNBC, was the only significant and independent prognostic marker in the node-negative patients [10].

Contrary to our supposition, our data in multivariate analysis showed that tumor subgroup (TNBC vs. non-TNBC) was not a prognostic factor for DFS. This study also revealed ER-positive and/or PgR-positive and HER2-negative patients had the best 5-year DFS, and the HER2-positive subtype showed the worst 5-year DFS. These findings were consistent with those of a previous study [6]. Meanwhile, our results contrast with those from other studies in terms of prognostic factors for DFS [3,4,12-14,20]. Liedtke reported that in multivariate analysis, a significantly decreased progression-free survival was observed for patients with TNBC compared with non-TNBC at the seventh year [3]. Yin reported that in multivariate analysis, TNBC had a significantly increased likelihood of recurrence within 2 years after surgery rather than thereafter [4]. Recent studies demonstrated that TNBC tends toward distant metastasis to the bone, soft tissue and viscera [3,10]. Liedtke reported that TNBC has a higher predilection for visceral metastasis and early recurrence within the first 3 years of follow-up [3]. Statistically, our study did not substantiate a higher predilection of TNBC factor for metastasis. In this study, there was no difference in DFS and a significant difference in overall survival in univariate analysis. This might indicate the difference in overall survival after recurrence. In our study, treatment after recurrence was based on NCCN or St. Gallen guidelines and multidisciplinary care discussions. Most patients who have a recurrence after breast conservation therapy were given completion mastectomy and adjuvant systemic chemotherapy.

In node-positive patients, our data supported the tendency of TNBC breast carcinoma to show a worse 5-year DFS, by log-rank analysis (Figure 2B). These results contrast with those from a previous study [10]. In node-negative patients, our results revealed that the four subgroups showed no difference in 5-year DFS or in 5-year overall survival. However, these results contrast with those from previous studies [10,21]. Rhee in Korea demonstrated that triple-negativity was an independent predictor of shorter relapse-free survival [21]. Table 1 in this study demonstrates that TNBC had a higher ratio of lymph node-negative patients than non-TNBC. This may explain why sometimes in our analysis the TNBC group was better than the HER2-positive subgroup.

However, the heterogeneity of TNBC, follow-up time, case number and other limitations may also explain the differences between our conclusions and those of previous studies. Research into the heterogeneity of TNBC, new neoadjuvant regimens and more molecular-based TNBC classification studies could give us more information about the optimal therapies for TNBC subgroups. Liedtke et al. followed their patients to the 7th year, Yin to the 11th year, and Cheang to the 15th years [3-5]. A longer follow-up period may yield different results.

In our study, the TNBC rate was 15.9%, which is similar to reports from Korea and Carolina [16,18], but lower than other reports [3-6,12,21]. The race factor could contribute to the difference. Carey also reported that the TNBC subtype has a higher incidence in pre-menopausal African-American women (39%), compared to non-African-American women of any age (16%) and post-menopausal African-American women (14%) [18].

This study revealed TNBC and non-TNBC patients displayed a similar distribution of clinico-pathological characteristics, such as age, tumor size, metastasis and stage, in contrast to other Western studies [3,10,15,19]. These previous reports indicated that TNBC was associated with a larger size, and therefore had a poorer outcome than non-TNBC in terms of DFS and overall survival. In our study, overall survival rates between these groups were statistically different in univariate analysis. This result may be related to lymph node, as mentioned. On the other hand, a higher grade was observed in our TNBC patients. In spite of this finding, the DFS for the TNBC subgroup was not statistically different from that of the non-TNBC subgroup, in contrast to earlier studies [3,6,17].

In our study, all of the data inferred that HER2 status added prognostic information for hormone receptor-positive breast cancer patients, confirming that luminal B tumors constituted a poor outcome compared to luminal A tumors, in accordance with other studies [5,6,22]. In Taiwan, adjuvant trastuzumab targeted therapy was rarely used before 2006 in the HER2-positive subtype, due to the medical insurance policy, which may have contributed substantially to the worst overall survival.

Some research about epidermal growth factor receptor (EGFR) is interesting, and may provide another possible answer for the question of TNBC prognostic value [5,11,23]. Cheang reported that the expanded surrogate of PgR, HER2, EGFR, and cytokeratin 5/6 indicate a more specific definition of basal-like breast cancer, mostly TNBC, which better predicts breast cancer survival [5]. Cheang also showed that the core basal group has 1.62 times greater risk for breast cancer-specific death, whereas the non-core basal group does not have a clinically significant risk [5]. Siziopikou reported that because the majority of TNBC patients express EGFR, the subgroup may derive benefit from EGFR-targeted therapies [11].

## Conclusion

In conclusion, our study indicates that TNBC tends to display a worse clinical course. Notably in node-positive patients, TNBC does play a prognostic role in overall survival. In Taiwan, new strategies of chemotherapy and targeted therapy should be investigated for patients with TNBC.

## Competing interests

The authors declare that they have no competing interests.

## Authors' contributions

CL designed the concept of this study, drafted the manuscript and performed treatment. HST, LSC and SJK collected the data and performed the statistical analysis. TWC approved the final manuscript. DRC designed the concept of this study and provided treatment coordination. All authors read and approved the final manuscript.

## Pre-publication history

The pre-publication history for this paper can be accessed here:

http://www.biomedcentral.com/1471-2407/9/192/prepub
